# Reproducible and scalable purification of extracellular vesicles using combined bind-elute and size exclusion chromatography

**DOI:** 10.1038/s41598-017-10646-x

**Published:** 2017-09-14

**Authors:** Giulia Corso, Imre Mäger, Yi Lee, André Görgens, Jarred Bultema, Bernd Giebel, Matthew J. A. Wood, Joel Z. Nordin, Samir EL Andaloussi

**Affiliations:** 1 Department of Laboratory Medicine, Karolinska Institutet, Stockholm, Sweden; 2 Department of Physiology, Anatomy and Genetics, University of Oxford, Oxford, United Kingdom; 3 Institute for Transfusion Medicine, University Hospital Essen, University of Duisburg-Essen, Essen, Germany; 4 Department of Chemistry and Biochemistry, University of Colorado, Colorado Springs, USA; 5 Institute of Technology, University of Tartu, Tartu, Estonia; 6 Evox Therapeutics, King Charles House, Park End Street, Oxford, United Kingdom

## Abstract

Extracellular vesicles (EVs) play a pivotal role in cell-to-cell communication and have been shown to take part in several physiological and pathological processes. EVs have traditionally been purified by ultracentrifugation (UC), however UC has limitations, including resulting in, operator-dependant yields, EV aggregation and altered EV morphology, and moreover is time consuming. Here we show that commercially available bind-elute size exclusion chromatography (BE-SEC) columns purify EVs with high yield (recovery ~ 80%) in a time-efficient manner compared to current methodologies. This technique is reproducible and scalable, and surface marker analysis by bead-based flow cytometry revealed highly similar expression signatures compared with UC-purified samples. Furthermore, uptake of eGFP labelled EVs in recipient cells was comparable between BE-SEC and UC samples. Hence, the BE-SEC based EV purification method represents an important methodological advance likely to facilitate robust and reproducible studies of EV biology and therapeutic application.

## Introduction

Extracellular vesicles (EVs) are nanosized cell-derived vesicles^1–3^ delimited by a lipid bilayer and typically divided into three subgroups, according to their biogenesis pathways; exosomes, microvesicles (MVs) and apoptotic bodies^4^. In this article, the term EVs will refer to exosomes and MVs only. Exosomes are 70–150 nm in size and originate from the endocytic pathway^5^ whereas MVs are generally larger, 100–1000 nm in diameter and bud directly from the plasma membrane^6, 7^. They carry proteins and RNAs, both miRNAs and mRNAs, and have been shown to transfer their cargo to recipient cells^3, 8, 9^. EVs are of fundamental importance in conveying critical intercellular messages^8, 10^ both in physiological and pathological processes, such as taking part in the coagulation cascade^11^, immune response^12–14^ as well as aiding the spread of malignancies^9, 15^ and viral infections^16, 17^.

Because of their small size, physicochemical properties and the complexity of the surrounding fluid, purification of EVs is a great challenge. The gold standard in the field is to purify EVs by sequential centrifugation followed by an ultracentrifuge (UC) step to pellet the EVs at 110,000 × *g* ^18^. We and others have previously shown that the UC step damages the vesicles and leads to aggregation^18–20^, which can ultimately affect downstream analysis^21^ or application of EVs^19, 22^. Furthermore, this technique is time consuming and prone to variable results due to the diverse protocols and equipment used in different laboratories^23^. To overcome these issues, several other promising purification techniques have been proposed, such as precipitation kits^24, 25^ and size exclusion chromatography (SEC)^19, 26^.

In this article, we have evaluated a novel liquid chromatography technique for EV purification: using core bead chromatography. The technology combines both size separation with bind-elute chromatography (BE-SEC) where large molecules bypass these beads while molecules smaller than 700 kDa penetrate the inert outer shell and bind to hydrophobic and positively charged octylamine ligands within the core. We hypothesised that the BE-SEC column would be suitable for EV purification from cell culture conditioned medium (CM). Since EVs are larger than 700 kDa, they would be eluted directly in the flow through, while small soluble proteins and impurities less than 700 kDa would enter the core and remain trapped. We show that the BE-SEC method is suitable for purification of EVs, with yields consistently reaching 80% and vesicular purity comparable to the gold standard method in the field. Herein, we propose a novel BE-SEC based purification of EVs which is fast, reliable and scalable.

## Results

### EV isolation with bind-elute size exclusion columns and characterization

Harvesting EVs with conventional protocols, such as sequential centrifugation, has raised questions concerning the intactness and the purity of the EVs after purification. Thus, novel methods are required to deplete unwanted molecules and maintain the integrity of the vesicles. Here, we tried to address these issues by using a commercially available chromatography column to isolate EVs. The beads used in the BE-SEC column are designed to trap molecules smaller than 700 kDa and allow larger particles to pass through. Hence, we postulated that the BE-SEC column would capture smaller soluble protein/impurities while allowing EVs to bypass the beads due to their relatively large size.

**Figure 1:**
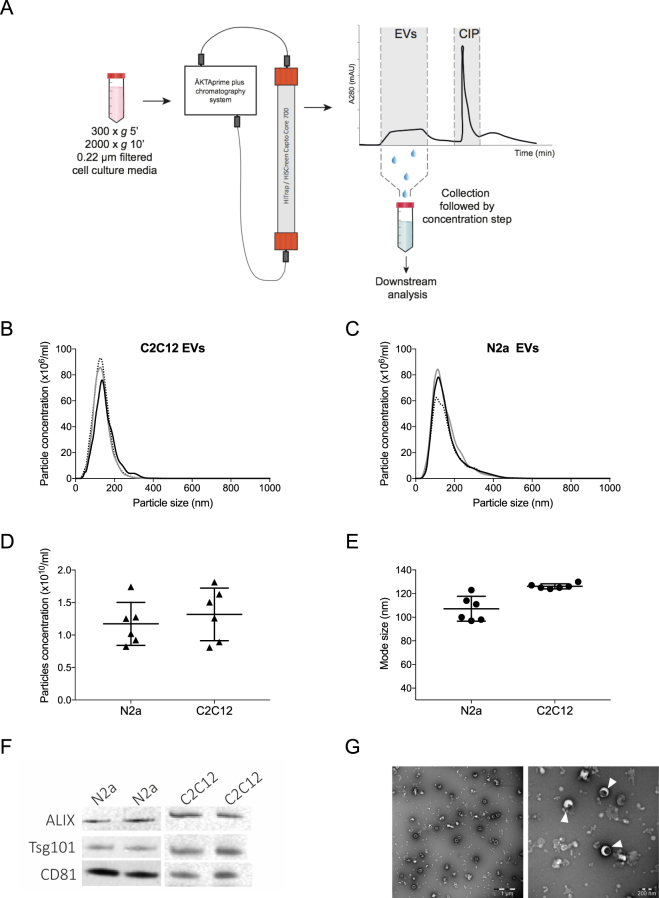
Characterization of neuroblastoma (N2a) and myoblast (C2C12) cell culture derived-EVs isolated with the BE-SEC column. (**A**) Schematic overview of the workflow. Processed CM was concentrated and loaded onto a BE-SEC column using the ÄKTA chromatography system. The first eluting fraction (EVs) was collected and subsequently concentrated and analysed. Contaminants that were trapped by the resin were eluted during the column wash (CIP) performed with 1 M NaOH in 30% isopropanol. (**B**,**C**) Representative particle concentrations and average size distributions of EVs derived from mouse N2a and C2C12 cell lines (n = 3). To assess the reproducibility of the method, the particle concentration (**D**) and the mode size (**E**) of independent experiments (n = 6) were plotted. (**F**) Western blotting analysis of BE-SEC purified vesicles derived from N2a and C2C12 (1 × 10^10^ particles loaded per well) was performed in duplicate. Full-length blots can be found in Supplementary Fig. 1. (**G**) TEM images of BE-SEC isolated EVs showing a wide field (left panel, scale bar 1 µm) and a close-up/zoomed-in picture (right panel, scale bar 200 nm). White triangles label EVs.

To test our hypothesis, conditioned medium (CM) from two different mouse cell sources was tested: neuroblastoma N2a, which we used in a previous study^19^ and myoblast C2C12 as comparison. Collected CM was subjected to two low speed spins and a 0.22 µm filtration step prior to purification on the BE-SEC column as illustrated in Fig. 1A. The size distribution and concentration of the BE-SEC isolated vesicles were evaluated by NTA (Fig. 1B–E). Particle size distribution and concentration of isolated vesicles ± SD were similar between six replicates (N2a 1.17 ± 0.33 × 10^10^ particles/ml, C2C12 1.32 ± 0.41 × 10^10^ particles/ml, Fig. 1D). Similarly, the particle mode size was constant within the same cell-derived EVs and in line with the expected EV size range (N2a 107 ± 10 nm, C2C12 126 ± 2 nm, Fig. 1E).

Moreover, the particle recovery rate compared to the input material (CM after the two low speed spins and 0.22 μm filtration), corresponded to 78.7 ± 17.3% for N2a and 73.8 ± 21.4% for C2C12, corroborating the high reproducibility of the procedure. To further characterize the vesicles, immunoblotting was performed and the EV markers Alix, Tsg101 and CD81 were detected on biological replicates (Fig. 1F). Additionally, transmission electron microscopy (TEM) showed intact cup shaped membrane vesicles with a size corresponding to the NTA results in all samples analysed (Fig. 1G and Supplementary Fig. 1). The purity of the vesicles isolated with the BE-SEC columns was also assessed based on particles/µg of protein (P/µg) according to Webber *et al*.^27^ (Table 1).

**Table 1:** Particles/µg of proteins.

	N2a		C2C12	
	Mean	SD	Mean	SD
CM	5.65e + 07	4.98e + 06	1.64e + 07	3.03e + 06
BE-SEC	2.05e + 09	5.89e + 08	2.10e + 09	1.88e + 08
UC	2.96e + 09	8.24e + 08	2.99e + 09	4.84e + 08

Purity index of vesicles in CM, BE-SEC and UC samples.

### BE-SEC can be utilised for processing large volumes of media

**Figure 2:**
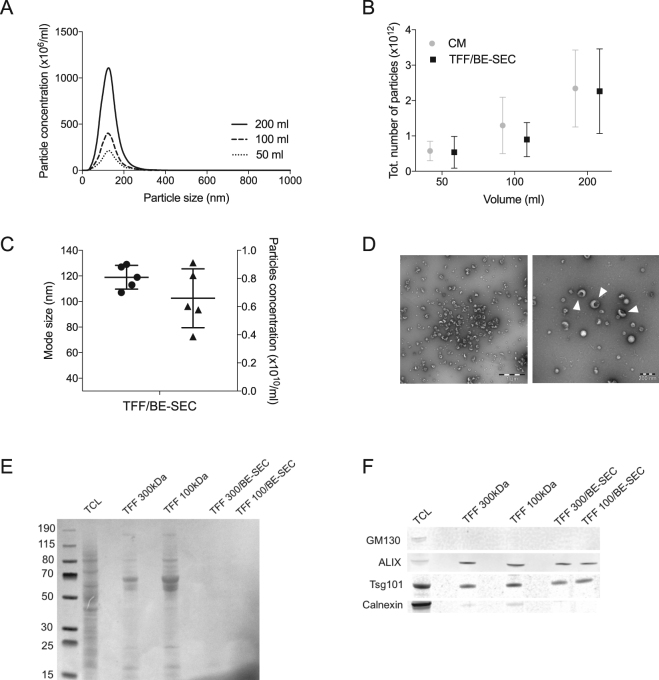
Validating the performance of TFF/BE-SEC in scaled-up experiments. (**A**) NTA analysis of TFF/BE-SEC N2a derived EVs isolated from different media volumes. (**B**) To assess the scalability, the total number of isolated particles was plotted against the different CM volumes tested. (**C**) Mode size (dots) and the particle concentration (triangles) of independent experiments (n = 5) were plotted to validate the reproducibility of the scaled-up samples. (**D**) Left, wide-field and right, close-up electron microscopy pictures of TFF/BE-SEC isolated EVs (white triangles pointing at EVs, scale bars left 1 µm and right 200 nm). (**E**) Total protein staining of cell lysate (TCL), TFF and TFF/BE-SEC samples. (**F**) WB analysis of N2a cells and N2a EVs (1 × 10^10^ particles per well) isolated with TFF (300 kDa and 100 kDa cut-off) and TFF/BE-SEC (TFF: Tangential Flow Filtration, TFF/BE-SEC: Tangential Flow Filtration coupled with bind-elute size exclusion chromatography). Full-length blots can be found in Supplementary Fig. 3.

The ability of the BE-SEC method to purify EVs from larger volumes of CM (50 ml up to 200 ml) was then investigated. To reduce the risk of loading the BE-SEC column with excessive amount of impurities that would exceed its binding ability (13 mg of ovalbumin/ml of medium), the samples were diafiltrated and concentrated using Tangential Flow Filtration (TFF). We have previously shown that 100 kDa spin filters are suitable for concentrating CM^19^ and therefore 100 kDa TFF filters were chosen for the concentration and diafiltraton step. After TFF, the concentrated CM was loaded onto a BE-SEC column (TFF/BE-SEC) for further purification. EVs were isolated from sequentially larger amounts of CM and analysed by NTA (Fig. 2A), which indicated that the increase in particle numbers was consistent with the increasing media volumes (Fig. 2B). Immunoblotting was performed to further characterise the vesicles. All EV markers tested, i.e. Alix, Tsg101, CD9 and CD81, were detected (Supplementary Fig. 2A and ﻿2﻿B). TEM (Fig. 2D) showed intact cup-shaped vesicles, morphologically similar to that in Fig. 1G and similar in size to those detected by NTA (Fig. 2A and ﻿2C). The TFF systems have a broad range of different filter sizes, hence a larger cut-off filter (300 kDa cut-off hollow-fibre filter unit) was evaluated as well. In theory, the larger cut-off would lead to a greater loss of protein impurities, although with the possible negative side effect of losing vesicles in the process. Indeed, both filter types retained over 95% of the particles and removed 80–90% of the proteins. Thereafter, total proteins were stained (Ponceau S staining) to evaluate the protein content in the samples after TFF and TFF/BE-SEC isolation. As expected more proteins were retained in the 100 kDa cut-off TFF filter compared to the 300 kDa filter and considerably less proteins and impurities were detected in the BE-SEC isolated samples regardless of the size of the TFF filter used (Fig. 2E). Nevertheless, the EV markers Alix and Tsg101 were equally detected among the samples, whereas Golgi and ER derived contaminants such as GM-130 and calnexin were only detected in the cell lysate and in the TFF-isolated samples (Fig. 2F and Supplementary Fig. 3). Furthermore, the P/µg ratio (Table 2) corroborated the total protein staining (Fig. 2E) with the 300 kDa filter removing notably more protein impurities compared to the 100 kDa filter. However, the P/µg values after BE-SEC processing were similar and in accordance with the values in Table 1.

**Table 2:** Particles/µg of proteins in CM, TFF and concentrated TFF/BE-SEC samples.

	N2a	
	Mean	SD
CM	2.32e + 07	4.95e + 05
TFF 100	4.50e + 08	2.24e + 08
TFF 100/BE-SEC	2.85e + 09	4.92e + 08
TFF 300	7.66e + 08	3.81e + 08
TFF 300/BE-SEC	3.25e + 09	3.20e + 08

### BE-SEC enables the removal of non-vesicular proteins and RNAs

One of the main issues of EV isolation is the discrimination between vesicular and non-vesicular secreted material, which may introduce a bias in downstream analysis. Thus, it was assessed whether the BE-SEC columns could provide such separation. To analyse the vesicular and non-vesicular fraction, media concentrated by TFF and TFF/BE-SEC were subjected to analytical SEC. Unlike BE-SEC, analytical SEC allows sample fractionation by size, without trapping smaller particles^19^. Using this technique, we aimed to verify the efficiency in removing smaller soluble proteins/impurities and RNAs by the prior TFF and BE-SEC steps.

**Figure 3:**
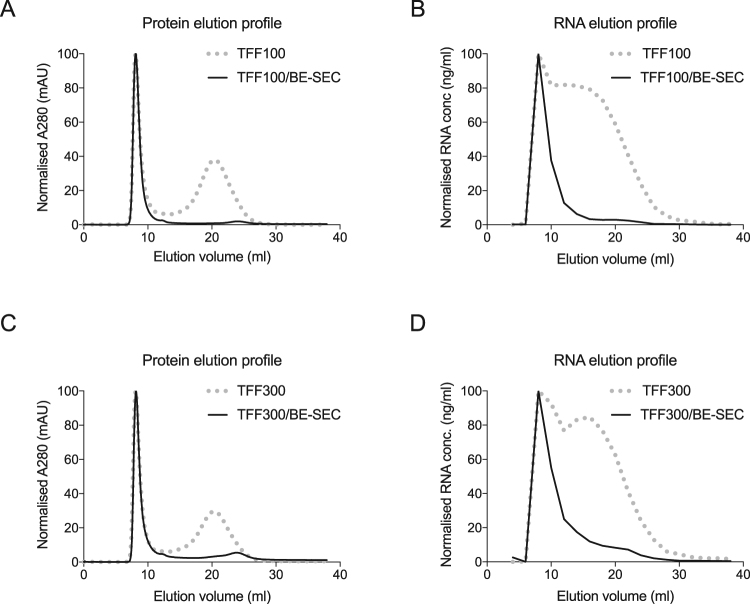
Depletion of non-vesicular proteins and RNAs. SEC analysis shows that non-vesicular proteins (**A–C**) and RNAs fraction (**B–D**) present in the TFF samples, are removed by the BE-SEC column (TFF100 or TFF300: Tangential Flow Filtration using 100 kDa or 300 kDa cut-off filters, TFF100/BE-SEC or TFF300/BE-SEC: Tangential Flow Filtration followed by bind-elute size exclusion chromatography).

The TFF isolated material, showed 2 distinct peaks; the first peak corresponding to the vesicular peak and the second peak representing the smaller non-vesicular ﻿proteins/impurities^19^. In contrast, SEC analysis of TFF/BE-SEC purified media, revealed the EV peak, whereas the non-vesicular protein peak was not detectable, regardless of the TFF cut-off filters used (Fig. 3A and ﻿3﻿C). Interestingly, when the RNA concentrations were measured, non-vesicular RNA fractions were strongly reduced following BE-SEC purification, but not upon TFF fractionation (Fig. 3B and 3﻿D).

### BE-SEC purified vesicles are taken up by recipient cells

**Figure 4:**
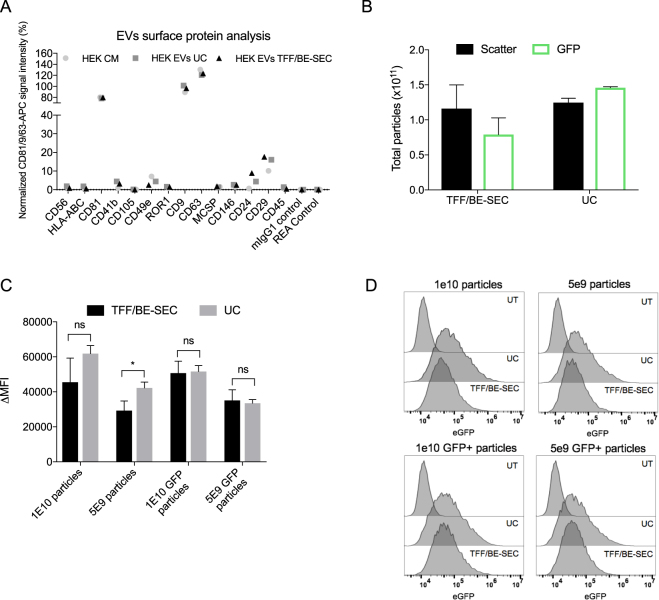
EV surface protein profile and EV uptake analysis by flow cytometry. (**A**) Signal intensity of respective bead populations normalized to the EV markers CD81/CD9/CD63. (**B**) NTA total particle count of scatter and fluorescent EVs isolated with UC and TFF/BE-SEC. (**C**) Mean fluorescence intensity normalized over the control (∆MFI) comparing the two isolation methods (n = 2). (**D**) Representative overlaid histograms of UC and TFF/BE-SEC isolated EVs uptake assay on recipient Huh7 cells, compared to untreated Huh7 cells (UT).

Surface protein composition of EVs has been shown to be important for the biodistribution^28^ and uptake of EVs^29, 30^, hence the surface protein profile of BE-SEC and UC purified vesicles was further investigated. To this end, the MACSPlex Exosome kit was used, which is composed of EV-capturing multiplexed beads coated with 37 different specific antibodies directed against epitopes mostly found in plasma or immune-cells derived vesicles^31^. For the detection by flow cytometry, the bead-bound EVs are counterstained with a mix of fluorophore-labelled antibodies directed against the EV markers CD81, CD9 and CD63^31^. To avoid EV concentration dependent effects in the measurements, wild-type HEK-293T derived EVs isolated by TFF/BE-SEC and UC, were re-diluted to the same particle concentration of the input material and analysed with the MACSPlex kit. The profile of EV surface epitopes was comparable to the CM and among samples regardless of the isolation method utilised (Fig. 4A). Although the kit was primarily designed for the detection of plasma and immune-cells derived EVs, we were able to detect 7 out of 37 surface markers among which the commonly used EV markers CD9, CD63 and CD81 were detected in high abundance.

Despite the comparability of the EV surface phenotype, molecules loosely associated with the EV surface might get lost during purification, eventually changing the EVs ability to bind to their selected target and affect their subsequent uptake into these cells. Thus, the cell uptake of CD63-eGFP labelled HEK-293T EVs purified either with UC or TFF/BE-SEC was quantified. Particle concentration was quantified by NTA both in light scatter and fluorescent mode (Fig. 4B). This allowed us to calculate the percentage of eGFP-positive particles in the isolated samples by comparing it to the total number of particles contained in the same sample; the TFF/BE-SEC samples had 68% eGFP-positive particles reflecting the eGFP percentage measured in the CM (data not shown), whereas the UC samples calculated fraction was 117%.

The higher eGFP percentage in the UC samples may be related to the physical changes that the high-speed centrifugation step causes to the vesicles e.g. protein/vesicles aggregation that may bias the particle counts and influence downstream analysis^20, 21^. To increase the specificity of the read-out, two doses of EVs were added to recipient cells based on both light scatter and ﻿eGFP NTA results (1 × 10^10^ and 5 × 10^9^ light scatter particles and ﻿eGFP particles) and incubated at 37 °C for 2 h. Internalization of CD63-eGFP EVs to recipient cells was evaluated using flow cytometry by analysing mean fluorescence intensity normalized over the control (∆MFI). EVs were taken up in a dose-dependent manner regardless of the isolation procedure (Fig. 4C and Supplementary Fig. 4) and as previously described^32–34^, 4 °C incubation blocked the vesicular uptake (data not shown), confirming that EV uptake is an energy dependent process. The higher percentage of eGFP positive particles detected in the UC isolated samples compared to the TFF/BE-SEC, was reflected in a higher fluorescent intensity signals when the total number of particles was taken into account. On the other hand, no significant differences were detected among differently isolated samples once the same amount of eGFP positive particles were applied (Fig. 4C and ﻿4D).

In summary, and taking a certain degree of variability into consideration, we could not detect any consistent difference concerning the EVs surface signature or their uptake after isolation with both methods.

## Discussion

The purification of EVs is a challenging endeavour due to their small size. Currently, a plethora of different isolation methods has been described but a standardized method is still missing. Ultracentrifugation is the most used purification protocol in the EV field^35^, but it has some limitations such as vesicular disruption, aggregation, limited processivity and operator-dependence^20, 22^. These issues have arisen concerns in the field, doubting the functionality of UC isolated EVs and therefore their effectiveness in a therapeutic context^21, 22, 36^.

Here, we describe an EV isolation technique (BE-SEC) previously utilised to purify viruses^37, 38^ similar in size to EVs^39^ and provide a comprehensive characterization of the isolated vesicles. Based on our data, the BE-SEC approach can purify vesicles with typical EV morphology and size, as judged by TEM and NTA, carrying common EV markers such as Alix, Tsg101 and CD81. The presence of typical EV proteins such as CD9, CD63 and CD81 was further detected using the MACSPlex assay system, corroborating the WB results. Moreover, the BE-SEC purification method provides isolation of non-fused and intact vesicles, in line with previously described SEC methods^19^.

Another limiting factor in the field is the large-scale production of EVs for therapeutic approaches^22^, therefore one of our aims was to test if the BE-SEC method was suitable for EV purification of large amounts of conditioned medium. The scalability of the method was investigated by concentrating and diafiltrating different cell culture media volumes with TFF followed by a BE-SEC clean-up step. Moreover, to increase the purity of the isolated vesicles, a comparison of two different TFF filter cut-offs (100 and 300 kDa) was evaluated.

Both filter types retained most of the vesicles regardless of the starting volume, although the 300 kDa hollow fibre was more efficient in clearing the sample of additional proteins prior to the BE-SEC clean-up. Nevertheless, after the BE-SEC isolation step the vesicular purity was relatively similar irrespective of the cut-off used. Since both filters retained nearly all vesicles and even though, under the conditions used in this study the cut-off did not impact the final purity, we propose the 300 kDa TFF hollow fibres as more suitable for EV purification. However, in case EV purification from larger volumes or more protein-rich media such as pre-spun is needed, cut-offs above 300 kDa are recommended. TFF hollow fibres with 750 kDa cut-offs are ideal to clear as much proteins as possible prior the final step, with yields similar to the above-mentioned cut-offs (unpublished data).

Circulating and cell-culture derived miRNAs have recently been shown to be released in the extracellular environment through vesicles^8^, however according to some studies such miRNAs are complexed with RNA binding proteins including Argonaute (AGO)^40, 41^. Evidence of EV mediated therapeutic effects driven by miRNAs is increasing and therefore the necessity of discriminating between vesicular and non-vesicular RNAs.

Hence, we tested whether the BE-SEC isolation method had the capability of capturing vesicular-free RNAs. According to analytical SEC fractionation and WB analysis, extra-vesicular RNA as well as soluble contaminating proteins were undetectable in the BE-SEC isolated EV samples. Finally, possible changes on the surface of the EVs caused by the BE-SEC purification were evaluated since the EV biodistribution and cellular uptake have been shown to be affected by the isolation method^28^ and EV surface alterations^29, 30^. However, TFF/BE-SEC isolated HEK-293T EVs showed surface marker profiles identical to the unprocessed starting material, suggesting that the isolation method does not disturb the natural EV surface signature.

Analogously, the uptake assay showed that HEK-293T:CD63-eGFP derived EVs were taken up by recipient cells to a similar extent regardless of the isolation method used. As an evidence of what has been previously described, regarding the vesicles integrity upon UC isolation^18, 20^, we observed that, in the UC samples, the amount of detected eGFP positive particles was higher than the total particles (NTA light scatter) of around 20%. Thus, we hypothesised that such signal could be due to the presence of fluorescent protein aggregates which cannot be distinguish from vesicles by the NTA^42^. In addition, the mode size of UC isolated sample decreased in the eGFP measurement corroborating our hypothesis (Supplementary Fig. 5).

In summary, we show that the BE-SEC columns can purify EVs in a reliable and scalable fashion with yields ranging from 70 to 80% and purity comparable to UC. Moreover, the BE-SEC method enables EV isolation in a time-efficient manner: from collection to analysis, the time ranges from 85 minutes for BE-SEC isolation, to 150 minutes in case of a prior concentration step using TFF.

Conclusively, there is an increased interest in tracking EVs *in vivo* and *in vitro*, engineering them with therapeutic cargos and moreover, isolate large-scale vesicles as therapies^22^. Therefore, based on our data, we propose that the BE-SEC method could possibly be used in the future as a clean-up step to remove unwanted proteins from previously concentrated large volumes of media or unbound dyes in the process of labelling EVs or unloaded cargos for therapeutic purposes. Therefore, the BE-SEC could perform at its best as a final clean-up step in the EV purification process.

## Material and Methods

### Cell culture

Mouse Neuroblastoma (N2a) and myoblast (C2C12) cells were seeded at a density of 4 × 10^6^ cells in 15 cm culture dishes in Dulbecco’s Modified Eagle’s Medium (DMEM) (Invitrogen), supplemented with 10% Fetal Bovine Serum (FBS) for N2a and 20% FBS for C2C12, 20 mM L-Glutamine and 1% penicillin (100 U/ml) and streptomycin (100 μg/ml) (P/S) and maintained at 37 °C, 5% CO_2_ atmosphere. After 24 h (cells reached a confluency of about 70%) the media was changed to OptiMEM reduced serum medium (Invitrogen) supplemented with 1% P/S followed by 48 h incubation before EV isolation. For the EV uptake experiment, HEK-293T cells stably expressing eGFP fused to the C-terminal of human CD63 (HEK-293T:CD63-eGFP, for more details please refer to supplementary information) were seeded at a density of 8 × 10^6^ cells in 15 cm cell culture dishes and cultured as stated above. According to the different cell lines characteristics, an adequate number of cells were seeded to reach 70–80% confluence on day 2.

### EV isolation using BE-SEC columns

CM was collected and subjected to a low speed spin at 300 × *g* for 5 minutes, followed by 2000 × *g* spin for 10 minutes to remove larger particles and cell debris. The supernatant was then filtered with a 0.22 μm syringe filter and subjected to different purification steps. Large volumes were diafiltrated and concentrated to roughly 20 ml using the Vivaflow 50 R tangential flow (TFF) device (Sartorius) with 100 kDa cut-off filters or the KR2i TFF system (SpectrumLabs) with 100 or 300 kDa cut-off hollow fibre filters at a flow rate of 100 ml/min (transmembrane pressure at 3.0 psi and shear rate at 3700 sec^−1^). The pre-concentrated CM was subsequently loaded onto the BE-SEC columns (HiScreen Capto Core 700 column, GE Healthcare Life Sciences), connected to an ÄKTAprime plus or ÄKTA Pure 25 chromatography system (GE Healthcare Life Sciences). Flow rate settings for column equilibration, sample loading and column cleaning in place (CIP) procedure were chosen according to the manufacturer’s instructions. The EV sample was collected according to the 280 nm UV absorbance chromatogram and concentrated using an Amicon Ultra-15 10 kDa molecular weight cut-off spin-filter (Millipore), washed with 30 ml PBS, concentrated to a final volume of 100 μl and stored at −80 °C for further downstream analysis. To assess the protein and RNA elution profiles, CM was concentrated and diafiltrated with KR2i TFF system using 100 kDa and 300 kDa hollow fibre filters and samples analysed on a Tricorn 10/300 Sepharose 4 Fast Flow (S4FF) column (GE Healthcare Life Sciences). Another pool was run through the Capto Core 700 column first and then analysed on the S4FF column. Protein and RNA elution profiles have been graphed with GraphPad Prism v.7.0b software and normalized to express a percentage.

### Nanoparticle tracking analysis

Particle size and concentration of the samples were determined via nanoparticle tracking analysis (NTA)^43, 44^ using NanoSight NS500 equipped with NTA 2.3 analytical software and a 488 nm laser. Samples were diluted in PBS and analysed. Five 30 second videos were recorded per sample with a camera level of 13–14. Software settings for analysis were kept constant for every measurement (screen gain 10, detection threshold 7). For the detection of fluorescent particles, the command stage settings were changed to have a continuous flow and five 30 second videos were recorded with a camera level of 15–16. Software settings were changed to screen gain 10, detection threshold 4–5 and minimum track length to 5. Every sample was also measured in light scatter mode with a camera level of 13–14 and analysed with the same settings but detection threshold 7. The NTA measurement in flow mode were used to calculate the percentage of eGFP positive particles over the total number of scatter particles in the sample.

### Protein and RNA quantification

Protein quantities in samples were measured using the DC protein assay kit (Bio-Rad) according to the manufacturer’s instructions. RNA concentration was quantified using the Quant-iT RiboGreen RNA assay kit (Thermo Fisher Scientific) according to the manufacturer’s instructions.

### Western blotting

Western blotting (WB) was performed using the iBlot® system (Invitrogen, Life Technologies) according to the manufacturer’s instructions. Equal numbers of particles of each sample were mixed with sample buffer (0.5 M ditiothreitol (DTT), 0.4 M sodium carbonate (Na_2_CO_3_), 8% SDS and 10% glycerol) and heated at 65 °C for 5 min. The mixture was then loaded onto a NuPAGE® Novex® 4–12% Bis-Tris Protein Gel and ran at 120 V in NuPAGE® MES SDS running buffer for 2 h. The proteins on the gel were transferred to an iBlot nitrocellulose membrane (Invitrogen) for 7 min using the iBlot system. Membranes were blocked with Odyssey blocking buffer for 60 min at RT with gentle shaking. After blocking, the membrane was incubated overnight at 4 °C or 1 h at RT with primary antibody solution (1:1000 dilution for anti-CD9 [ab92726, Abcam], anti-Alix [ab117600, Abcam], anti-Tsg101 [ab30871, Abcam], anti-Calnexin [ab22595, Abcam], 1:500 dilutions for anti-GM130 [clone 35/GM130, BD Biosciences] and 1:200 dilution for anti-CD81 [sc-9158, Santa Cruz]). The membrane was washed with PBS supplemented with 0.1% Tween 20 (PBS-T) for 5 minutes, 5 times and incubated with the corresponding secondary antibody for 1 h at RT (1:15000 anti-mouse IgG DyLight-800 to detect Alix; 1:15000 dilution anti-rabbit IgG DyLight-800 to detect CD9, Tsg101, CD81 and Calnexin). Membranes were washed with PBS-T for 5 minutes 5 times, one time with PBS and visualized on the Odyssey infrared imaging system (LI-COR).

### Electron Microscopy

Purified EVs were added onto glow-discharged formvar-carbon type B coated electron microscopy grids (Ted Pella Inc). The grid was dried with filter paper and stained with 2% uranyl acetate in double distilled H_2_O (Sigma) for 10 seconds. After the stain was completed, the grid was washed with distilled water and blotted dry with filter paper. The grid was air dried and visualized using a transmission electron microscope (Tencai 10).

### EV surface protein profiling by flow cytometry

HEK-293T derived EVs were isolated with UC and BE-SEC and diluted to the original particles concentration detected in the CM. EV staining with the MACSPlex Exosome kit, human (Miltenyi Biotec) was performed at 4 °C overnight according to the manufacturer’s instructions. The samples were analysed with a Cytoflex S flow cytometer (Beckman Coulter) with at least 10,000 recorded events per sample. Data were analyzed with FlowJo software (version 10.0.7). The mean fluorescence values plotted in the graph were background corrected and normalized on CD63/81/9 mean signal intensity as previously described^31, 45^. Negative values were excluded from the plot.

### EV uptake assay using flow cytometry

For comparison, HEK-293T:CD63-eGFP derived EVs were isolated with UC (110,000 × *g* for 90 minutes followed by PBS wash) and TFF/BE-SEC as previously described. Particle concentration and size were analysed with NTA both in scatter and fluorescence mode. A fixed number of particles were added (1 × 10^10^ and 5 × 10^9^ particles based on NTA scatter and fluorescence mode) to human hepatocellular carcinoma cells (Huh-7) seeded the day before at a density of 7.5 × 10^4^ cells per well in a 24-well plate. Cells were incubated for 2 h at 37 °C, 5% CO_2_ atmosphere. After incubation, the cells were washed twice with PBS, collected, spun down at 300 × *g* for 5 minutes and resuspended in 100 µl of Dulbecco’s phosphate buffered saline (Invitrogen), 1 mM EDTA and 2% FBS. Dead cells were excluded from analysis via 4’,6-diamidino-2-phenylindole (DAPI) staining and doublets were excluded by forward/side scatter area *versus* height gating. Samples were kept on ice and measured with the Cytoflex S flow cytometer (Beckman Coulter). Data was analysed with the FlowJo software (version 10.0.7). Mean fluorescence intensity was normalized over the control/untreated cell sample (∆MFI). Statistical significance was evaluated with GraphPad Prism (version 7.0b). One unpaired student t-test was performed and a *P-value* < 0.05 was considered statistically significant.

### Data availability

The data generated during the current study are available from the corresponding author upon reasonable request.

## Electronic supplementary material

Supplementary Information

## References

[1] Raposo G, Stoorvogel W (2013). Extracellular vesicles: Exosomes, microvesicles, and friends. The Journal of Cell Biology.

[2] Fauré J (2006). Exosomes are released by cultured cortical neurones. Molecular and Cellular Neuroscience.

[3] Ratajczak J (2006). Embryonic stem cell-derived microvesicles reprogram hematopoietic progenitors: evidence for horizontal transfer of mRNA and protein delivery. Leukemia.

[4] El Andaloussi S, Mäger I, Breakefield XO, Wood MJA (2013). Extracellular vesicles: biology and emerging therapeutic opportunities. Nature reviews. Drug discovery.

[5] Pan BT, Teng K, Wu C, Adam M, Johnstone RM (1985). Electron microscopic evidence for externalization of the transferrin receptor in vesicular form in sheep reticulocytes. Journal of Cell Biology.

[6] György B (2011). Membrane vesicles, current state-of-the-art: Emerging role of extracellular vesicles. Cellular and Molecular Life Sciences.

[7] Holme P, Solum N, Brosstad F, Røger M, Abdelnoor M (1994). Demonstration of platelet-derived microvesicles in blood from patients with activated coagulation and fibrinolysis using a filtration technique and western blotting. Thrombosis and haemostasis.

[8] Valadi H (2007). Exosome-mediated transfer of mRNAs and microRNAs is a novel mechanism of genetic exchange between cells. Nature Cell Biology.

[9] Skog J (2008). Glioblastoma microvesicles transport RNA and proteins that promote tumour growth and provide diagnostic biomarkers. Nature Cell Biology.

[10] G Raposo HWNWSRLCVHCJM, Geuze HJB (1996). Lymphocytes Secrete Antigen-presentingVesicles. The Journal of Experimental Medicine.

[11] Del Conde I, Shrimpton CN, Thiagarajan P, López JA (2005). Tissue-factor-bearing microvesicles arise from lipid rafts and fuse with activated platelets to initiate coagulation. Blood.

[12] Admyre C, Johansson SM, Paulie S, Gabrielsson S (2006). Direct exosome stimulation of peripheral human T cells detected by ELISPOT. European Journal of Immunology.

[13] Segura E, Amigorena S, Théry C (2005). Mature dendritic cells secrete exosomes with strong ability to induce antigen-specific effector immune responses. Blood Cells, Molecules, and Diseases.

[14] Théry C (2002). Indirect activation of naïve CD4 + T cells by dendritic cell–derived exosomes. Nature Immunology.

[15] Peinado H (2012). Melanoma exosomes educate bone marrow progenitor cells toward a pro-metastatic phenotype through MET. Nat Med.

[16] Mack M (2000). Transfer of the chemokine receptor CCR5 between cells by membrane-derived microparticles: a mechanism for cellular human immunodeficiency virus 1 infection. Nature medicine.

[17] Rozmyslowicz T (2003). Platelet- and megakaryocyte-derived microparticles transfer CXCR4 receptor to CXCR4-null cells and make them susceptible to infection by X4-HIV. AIDS (London, England).

[18] Théry, C., Amigorena, S., Raposo, G. & Clayton, A. Isolation and characterization of exosomes from cell culture supernatants and biological fluids. *Current protocols in cell biology / editorial board*, *Juan S*. *Bonifacino… [et al*.*]* Chapter 3, Unit 3.22-Unit 23.22, doi:10.1002/0471143030.cb0322s30 (2006). 10.1002/0471143030.cb0322s30 18228490

[19] Nordin JZ (2015). Ultrafiltration with size-exclusion liquid chromatography for high yield isolation of extracellular vesicles preserving intact biophysical and functional properties. Nanomedicine: Nanotechnology, Biology, and Medicine.

[20] Linares R, Tan S, Gounou C, Arraud N, Brisson AR (2015). High-speed centrifugation induces aggregation of extracellular vesicles. J Extracell Vesicles.

[21] Van Deun, J. *et al*. The impact of disparate isolation methods for extracellular vesicles on downstream RNA profiling. *J Extracell Vesicles* **3**, doi:10.3402/jev.v3.24858 (2014). 10.3402/jev.v3.24858 PMC4169610 25317274

[22] Lener T (2015). Applying extracellular vesicles based therapeutics in clinical trials - an ISEV position paper. J Extracell Vesicles.

[23] Livshits MA (2015). Isolation of exosomes by differential centrifugation: Theoretical analysis of a commonly used protocol. Sci Rep.

[24] Kordelas L (2014). MSC-derived exosomes: a novel tool to treat therapy-refractory graft-versus-host disease. Leukemia.

[25] Ghosh A (2014). Rapid isolation of extracellular vesicles from cell culture and biological fluids using a synthetic peptide with specific affinity for heat shock proteins. PLoS One.

[26] Boing, A. N. *et al*. Single-step isolation of extracellular vesicles by size-exclusion chromatography. *J Extracell Vesicles* **3**, doi:10.3402/jev.v3.23430 (2014). 10.3402/jev.v3.23430 PMC4159761 25279113

[27] Webber J, Clayton A (2013). How pure are your vesicles?. Journal of extracellular vesicles.

[28] Wiklander OPB (2015). Extracellular vesicle *in vivo* biodistribution is determined by cell source, route of administration and targeting. Journal of extracellular vesicles.

[29] Mulcahy LA, Pink RC, Carter DRF (2014). Routes and mechanisms of extracellular vesicle uptake. Journal of extracellular vesicles.

[30] Hoshino A (2015). Tumour exosome integrins determine organotropic metastasis. Nature.

[31] Koliha N (2016). A novel multiplex bead-based platform highlights the diversity of extracellular vesicles. J Extracell Vesicles.

[32] Svensson KJ (2013). Exosome Uptake Depends on ERK1/2-Heat Shock Protein 27 Signaling and Lipid Raft-mediated Endocytosis Negatively Regulated by Caveolin-1. Journal of Biological Chemistry.

[33] Escrevente C, Keller S, Altevogt P, Costa J (2011). Interaction and uptake of exosomes by ovarian cancer cells. BMC Cancer.

[34] Barres C (2010). Galectin-5 is bound onto the surface of rat reticulocyte exosomes and modulates vesicle uptake by macrophages. Blood.

[35] Gardiner C (2016). Techniques used for the isolation and characterization of extracellular vesicles: results of a worldwide survey. J Extracell Vesicles.

[36] Mol, E. A., Goumans, M. J., Doevendans, P. A., Sluijter, J. P. & Vader, P. Higher functionality of extracellular vesicles isolated using size-exclusion chromatography compared to ultracentrifugation. *Nanomedicine*, doi:10.1016/j.nano.2017.03.011 (2017). 10.1016/j.nano.2017.03.011 28365418

[37] Blom H (2014). Efficient chromatographic reduction of ovalbumin for egg-based influenza virus purification. Vaccine.

[38] James KT (2016). Novel High-throughput Approach for Purification of Infectious Virions. Scientific reports.

[39] Nolte-‘t Hoen E, Cremer T, Gallo RC, Margolis LB (2016). Extracellular vesicles and viruses: Are they close relatives?. Proc. Natl. Acad. Sci. USA.

[40] Arroyo JD (2011). Argonaute2 complexes carry a population of circulating microRNAs independent of vesicles in human plasma. Proceedings of the National Academy of Sciences.

[41] Turchinovich A, Weiz L, Langheinz A, Burwinkel B (2011). Characterization of extracellular circulating microRNA. Nucleic Acids Res.

[42] Piffoux, M., Gazeau, F., Wilhelm, C. & Silva, A. K. In *Design and Applications of Nanoparticles in Biomedical Imaging* 43–68 (Springer, 2017).

[43] Sokolova V (2011). Characterisation of exosomes derived from human cells by nanoparticle tracking analysis and scanning electron microscopy. Colloids Surf B Biointerfaces.

[44] Dragovic RA (2011). Sizing and phenotyping of cellular vesicles using Nanoparticle Tracking Analysis. Nanomedicine.

[45] Koliha, N. *et al*. Melanoma Affects the Composition of Blood Cell-Derived Extracellular Vesicles. *Frontiers in Immunology* **7**, doi:10.3389/fimmu.2016.00282 (2016). 10.3389/fimmu.2016.00282 PMC4960424 27507971

