# Supplementary material for: Reproducible and scalable purification of extracellular vesicles using combined bind-elute and size exclusion chromatography
Source: Sci Rep. 2017 Sep 14;7:11561. doi: 10.1038/s41598-017-10646-x (PMC5599601; doi:10.1038/s41598-017-10646-x)
Supplement: Supplementary file 1 — Supplementary Information [file 41598_2017_10646_MOESM1_ESM.doc]

**Reproducible and scalable purification of extracellular vesicles using combined bind-elute and size exclusion chromatography**

Giulia Corso^1+^, Imre Mäger^2,5+^, Yi Lee^1^, André Görgens^1,3^, Jarred Bultema^4^, Bernd Giebel^3^, Matthew J.A. Wood^2,6^, Joel Z. Nordin^1,6*^ and Samir EL Andaloussi^1,2,6*^

^1^Department of Laboratory Medicine, Karolinska Institutet, Stockholm, Sweden

^2^Department of Physiology, Anatomy and Genetics, University of Oxford, Oxford, United Kingdom

^3^Institute for Transfusion Medicine, University Hospital Essen, University of Duisburg-Essen, Essen, Germany
^4^ Department of Chemistry and Biochemistry, University of Colorado,

Colorado Springs, USA

^5^Institute of Technology, University of Tartu, Tartu, Estonia

^6^Evox Therapeutics, King Charles House, Park End Street, Oxford, United Kingdom

**Supplementary Information**

*Establishment of the stable HEK293T:CD63-eGFP cell line*

The HEK293T:CD63-eGFP stable cell line was generated by lentiviral mediated gene transfer of human CD63 cDNA (GenBank accession number CR542096) fused to the N-terminus of eGFP. First, the coding region of CD63 without stop-codon was amplified via PCR from HEK293T cDNA by using the oligonucleotides 5’ACCGATCTCGAGCAATGGCGGTGGAAGGAGGAATG and 3’ACCGATGAATTCTCACCTCGTAGCCACTTCTGATACT, thereby introducing flanking XhoI/EcoRI restriction sites, and cloned into the peGFP-N1 plasmid (Takara Bio Europe/SAS). Next, the CD63-eGFP ORF was transferred to the lentiviral pCL6-IEGwo expression plasmid (kindly provided by Helmut Hanenberg, University Hospital Essen) via NheI/BsrGI restriction sites. The CD63-eGFP expression cassette of the resulting lentiviral plasmid pCL6-CD63eGFP was confirmed by sequencing.

Lentiviral supernatants were produced as described previously^46^. Briefly, HEK293T cells were co-transfected with the plasmids pCL6-CD63-eGFP, pCD/NL-BH and pcoPE^47-49^ using the transfection reagent JetPEI (Polyplus, Illkrich Cedex). 16 h post transfection, gene expression from the human cytomegalovirus (CMV) immediate-early gene enhancer/promoter was induced with 10 mM sodium butyrate (Sigma Aldrich) for 6-8 h before fresh medium was added to the cells, and the supernatant was collected 20 h later. Viral particles were pelleted at 25,000 xg for 90 minutes at 4˚C. The supernatant was discarded and the pellet was resuspended in 2 ml of Iscove’s Modified Dulbecco’s Media supplemented with 20% FBS and 1% P/S. Aliquots were stored at -80°C until usage. HEK293T cells were transduced by overnight exposure to virus stocks, passaged at least twice, and subsequently sort-purified (eGFP expression) on a BD FACSAria IIIu cell sorter. The established cell line was passaged at least 5 times before experiments were performed.

**Supplementary Figures**

**
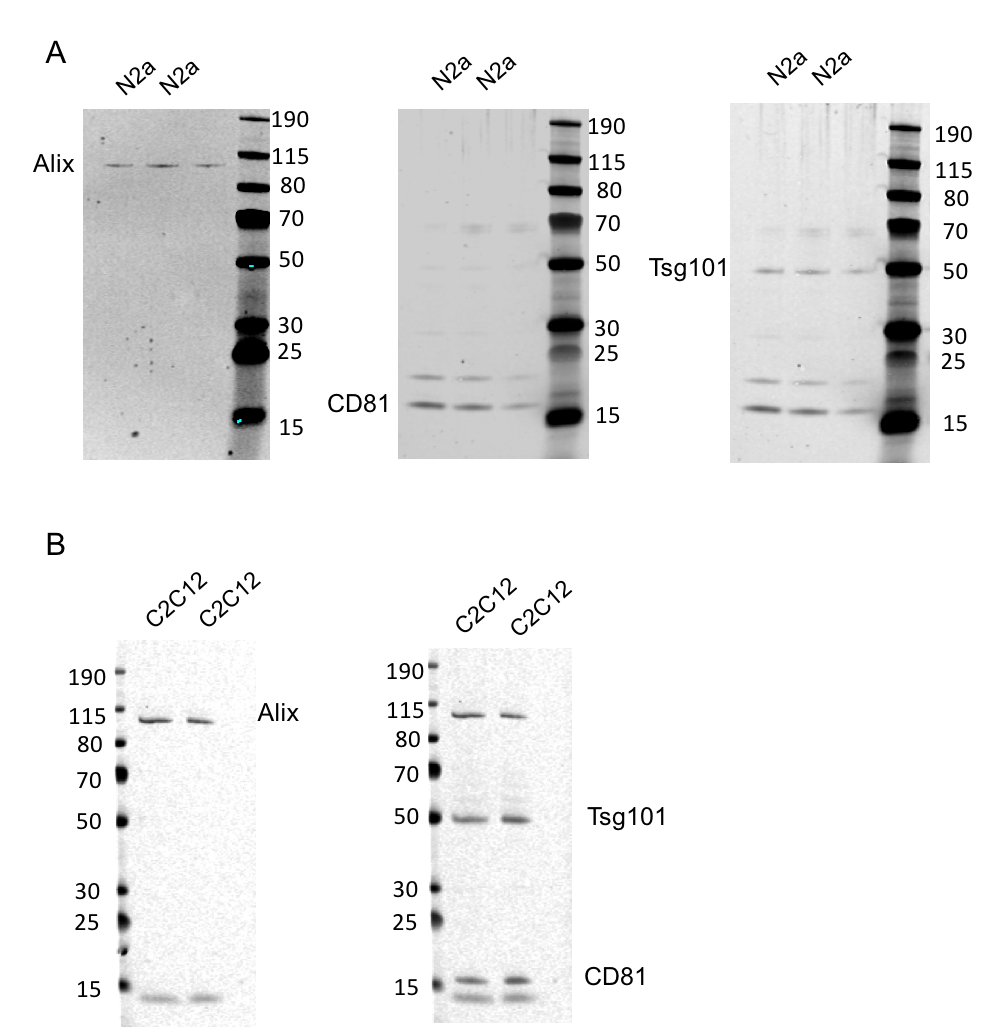
**

**Supplementary Figure 1.** Uncropped WB analysis of Figure 1F. **(A)** N2a-derived EVs and **(B)** C2C12-derived EVs immunoblot analysis.

**
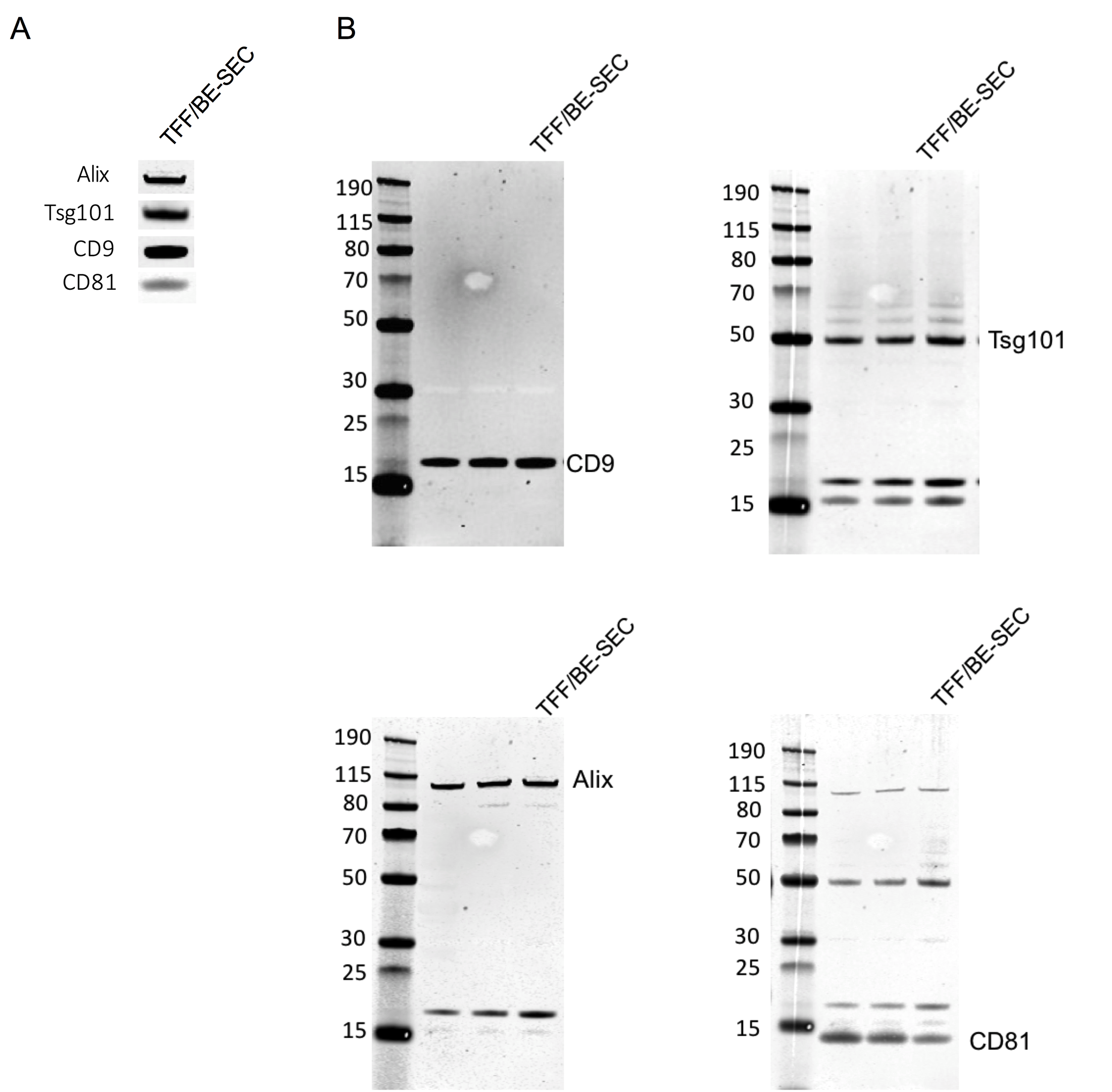
**

**Supplementary Figure 2.** **(A)** Western Blot (WB) analysis of TFF/BE-SEC purified vesicles (2.5x10^10^ particles per well). **(B)** Uncropped WB analysis of Supplementary Figure 2A.

**
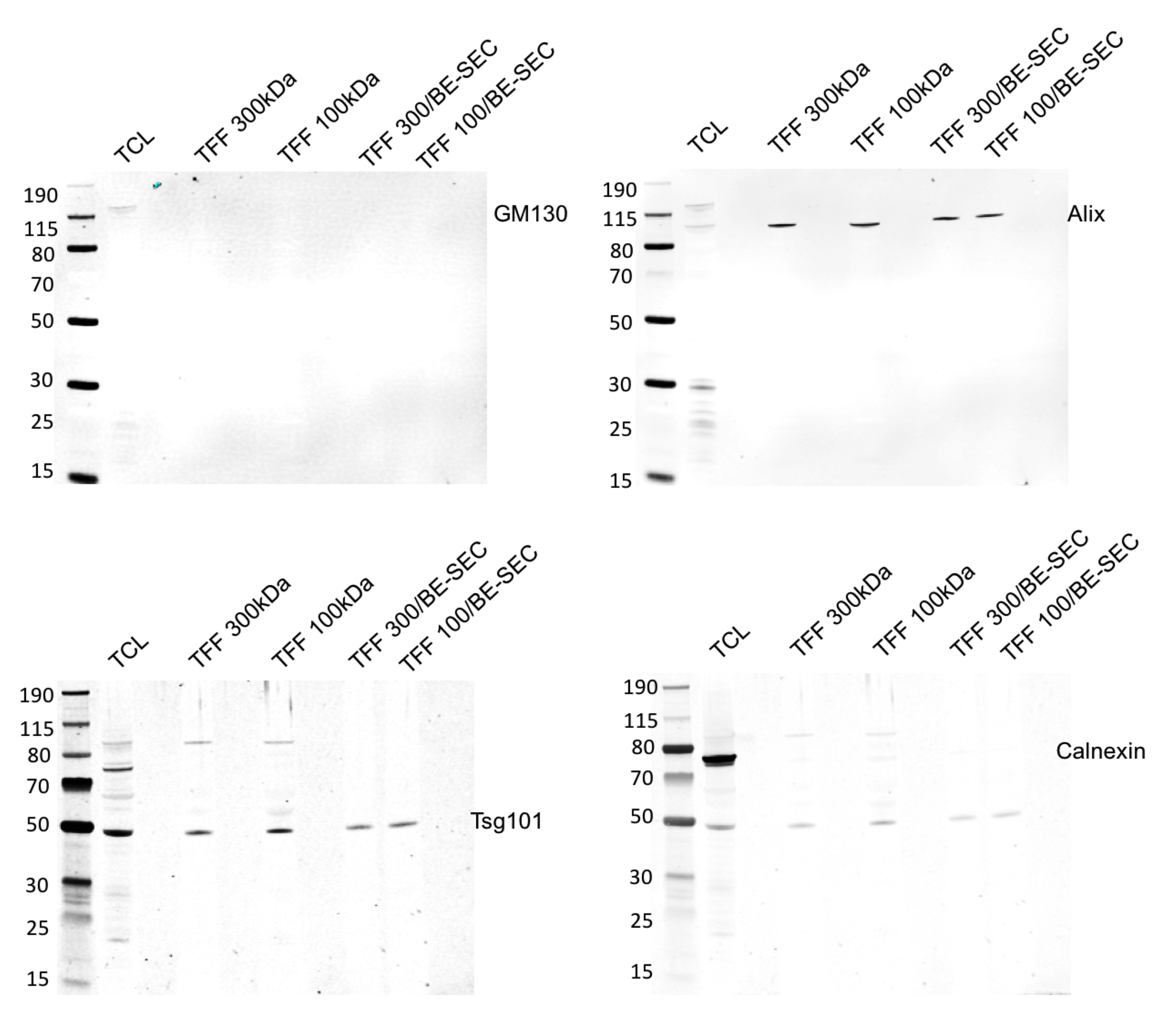
**

**Supplementary Figure 3.** Uncropped WB analysis of Figure 2F.


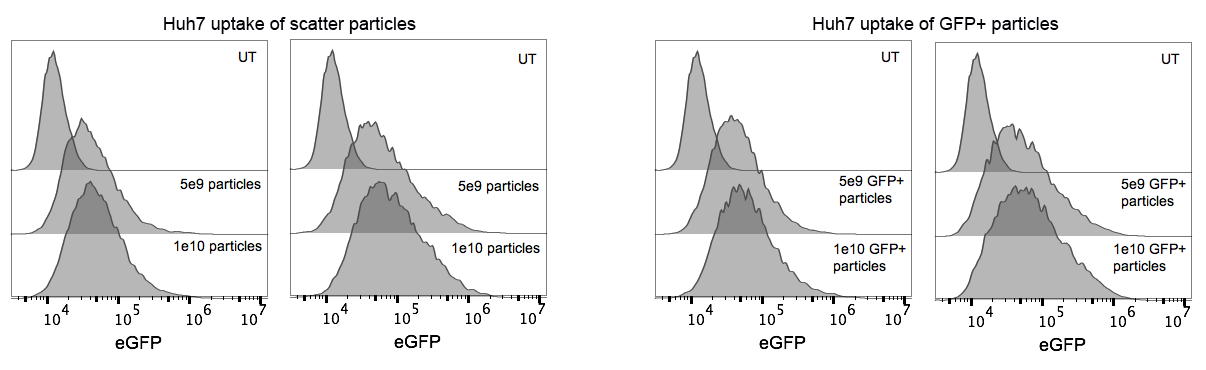


**Supplementary Figure 4. Dose-dependent EVs uptake on recipient cells.** Overlaid histograms of TFF/BE-SEC and UC purified samples. Different amounts of CD63-eGFP labelled-EVs **(**5x10^9^ and 1x10^10^ particles, measurements based on NTA light scatter mode) were added to recipient cells and internalization was quantified flow cytometrically.


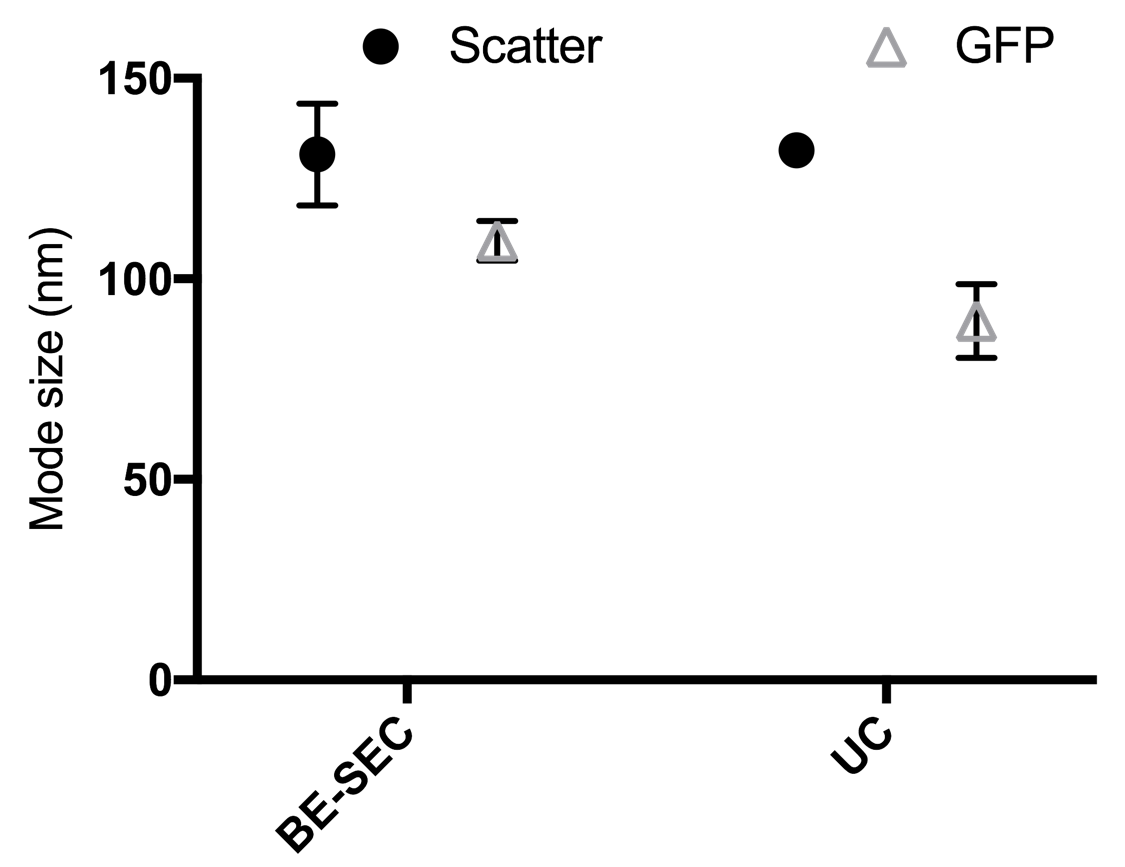


**Supplementary Figure 5. HEK293T:CD63-eGFP particle mode size.** NTA mode size analysis of BE-SEC and UC isolated HEK293T:CD63-eGFP EVs in light scatter and GFP mode.

**Supplementary References**

**46.** Gorgens, A. et al. Multipotent hematopoietic progenitors divide asymmetrically to create progenitors of the lymphomyeloid and erythromyeloid lineages. Stem Cell Reports 3, 1058–1072, doi:10.1016/j.stemcr.2014.09.016 (2014).

**47.** Mochizuki, H., Schwartz, J. P., Tanaka, K., Brady, R. O. & Reiser, J. High-titer human immunodeficiency virus type 1-based vector systems for gene delivery into nondividing cells. Journal of virology 72, 8873–8883 (1998).

**48.** Leurs, C. et al. Comparison of three retroviral vector systems for transduction of nonobese diabetic/severe combined immunodeficiency mice repopulating human CD34 + cord blood cells. Human gene therapy 14, 509–519, doi:10.1089/104303403764539305 (2003).

**49.** Müllers, E. et al. Novel functions of prototype foamy virus Gag glycine- arginine-rich boxes in reverse transcription and particle morphogenesis. Journal of virology 85, 1452–1463, doi:10.1128/JVI.01731-10 (2011).
